# Primary Cardiac Neoplasms: A 12-Year Institutional Experience at a National Cardiac Referral Center in Mexico

**DOI:** 10.7759/cureus.108263

**Published:** 2026-05-04

**Authors:** Arturo Bernal Armenta

**Affiliations:** 1 Department of Cardiothoracic Surgery, Unidad Médica de Alta Especialidad (UMAE) Hospital de Cardiología, Centro Médico Nacional Siglo XXI, Instituto Mexicano de Seguro Social, Mexico City, MEX

**Keywords:** cardiac myxoma, cardiac surgery, cardiac tumor prevalence, cardiac tumors, left atrial tumors, primary cardiac neoplasms

## Abstract

Background: Cardiac tumors are rare entities within cardiovascular pathology. Most primary cardiac neoplasms are benign and originate from mesenchymal tissue. Epidemiological data from tertiary referral centers remain limited in Latin America. Objective: This study aims to describe the prevalence and histological distribution of cardiac neoplasms resected at the Hospital de Cardiología, Centro Médico Nacional Siglo XXI, between 2012 and 2024. Materials and methods: A retrospective observational study was conducted. Medical records and histopathological reports of patients who underwent surgical resection of cardiac tumors between January 2012 and December 2024 were reviewed. Demographic characteristics, tumor location, and histological subtype were analyzed using descriptive statistics. Results: A total of 119 patients underwent surgical resection of cardiac tumors. The mean age at surgery was 56.7 ± 13.4 years, with a predominance of female patients (77.3%). The left atrium was the most frequent tumor location (84%). Histologically, cardiac myxoma was the most common neoplasm (92.4%), followed by papillary fibroelastoma (5.9%) and rhabdomyoma (0.8%). Only one malignant tumor (0.8%), corresponding to angiosarcoma, was identified. Conclusions: Cardiac tumors remain rare entities, with a predominance of benign mesenchymal neoplasms. In this single-center series, cardiac myxoma was the most frequent tumor and predominantly affected middle-aged women, with the left atrium as the most common location.

## Introduction

Primary cardiac tumors are rare clinical entities, with reported incidences ranging from 0.001% to 0.03% in autopsy series. Among these, benign tumors are more common than malignant neoplasms, with cardiac myxomas representing the most frequently diagnosed benign tumor in adults. These tumors typically arise in the left atrium and may present with obstructive, embolic, or constitutional symptoms depending on their size and location [1-3].

Approximately 75% of primary cardiac tumors are benign, with myxomas being the most common histological subtype. Malignant primary cardiac tumors are considerably less frequent but carry a significantly worse prognosis. Cardiac sarcomas constitute the majority of malignant cases and are often diagnosed at advanced stages due to their aggressive growth and nonspecific clinical presentation. Advances in imaging modalities such as echocardiography, computed tomography, and magnetic resonance imaging have improved the diagnostic evaluation of cardiac masses [4-7].

Due to the rarity of these tumors, most available data come from single-center retrospective studies. Large institutional experiences are valuable to better understand their clinical presentation, histopathological distribution, and surgical outcomes.

This study aims to describe the prevalence, demographic characteristics, anatomical distribution, and histopathological subtypes of primary cardiac neoplasms in patients who underwent surgical resection at our institution over a 12-year period.

## Materials and methods

Study design and population

This was a retrospective, descriptive, single-center study conducted at the Hospital de Cardiología, Centro Médico Nacional Siglo XXI, a national cardiology referral center in Mexico. Clinical records and histopathological reports of patients who underwent surgical resection of a cardiac tumor between January 2012 and December 2024 were reviewed. Only cases with histopathological confirmation of a primary cardiac neoplasm were included in the analysis.

Inclusion and exclusion criteria

Inclusion criteria were all patients who underwent surgical resection of a primary cardiac tumor with histopathological confirmation. Exclusion criteria included patients without histopathological confirmation, patients with metastatic cardiac tumors, and patients with incomplete clinical or pathological records.

Data collection and variables

Demographic, anatomical, and histopathological data were extracted from the available institutional records and entered into a database for analysis. The variables collected included age at the time of surgery, sex, tumor anatomical location, and histopathological subtype.

Data quality and missing data

The dataset was reviewed for internal consistency and errors before analysis. Missing or unavailable data were not imputed and were excluded from variable-specific analyses.

Statistical analysis

Descriptive statistical analysis was performed. Categorical variables are presented as frequencies and percentages. For continuous variables, normality was assessed using visual inspection of histograms and the Kolmogorov-Smirnov test. Variables with normal distribution are expressed as mean ± standard deviation, while non-normally distributed variables are reported as median and interquartile range. All analyses were performed using Microsoft Excel (Microsoft Corp., Redmond, WA, USA).

Ethical considerations

The study was approved by the Research and Ethics Committee, UMAE Hospital de Cardiología, Centro Médico Nacional Siglo XXI, Instituto Mexicano del Seguro Social (protocol number R-2025-3604-017). Patient confidentiality was protected throughout the study by anonymizing the collected data prior to analysis.

## Results

During the period between January 2012 and December 2024, clinical records and histopathological reports of patients who underwent surgical resection of a cardiac tumor were reviewed. A total of 119 cases with pathological confirmation were identified (Table 1).

**Table 1:** Summary of baseline characteristics of patients with primary cardiac neoplasms (n = 119) SD: standard deviation

Variable	Value
Total patients	119
Age, mean ± SD (years)	56.77 ± 13.48
Age range (years)	16-84
Most represented age group	60-69 years
Female sex	92 (77.3%)
Male sex	27 (22.7%)
Left atrium	100 (84.0%)
Right atrium	11 (9.2%)
Left ventricle	2 (1.7%)
Right ventricle	2 (1.7%)
Aortic valve	3 (2.5%)
Mitral valve	1 (0.8%)
Myxoma	110 (92.4%)
Papillary fibroelastoma	7 (5.9%)
Rhabdomyoma	1 (0.8%)
Angiosarcoma	1 (0.8%)

**Figure 1:**
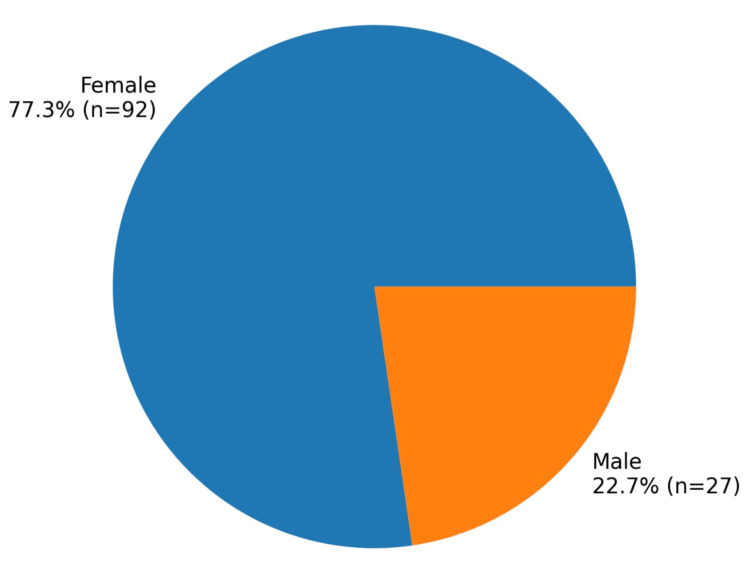
Distribution of patients by sex (n = 119)

Regarding sex distribution, female patients were predominant, with 92 women (77.3%) and 27 men (22.7%). The mean age at the time of surgery was 56.77 ± 13.48 years, ranging from 16 to 84 years. The most frequently represented age group was 60 to 69 years (Figure 1).

**Figure 2:**
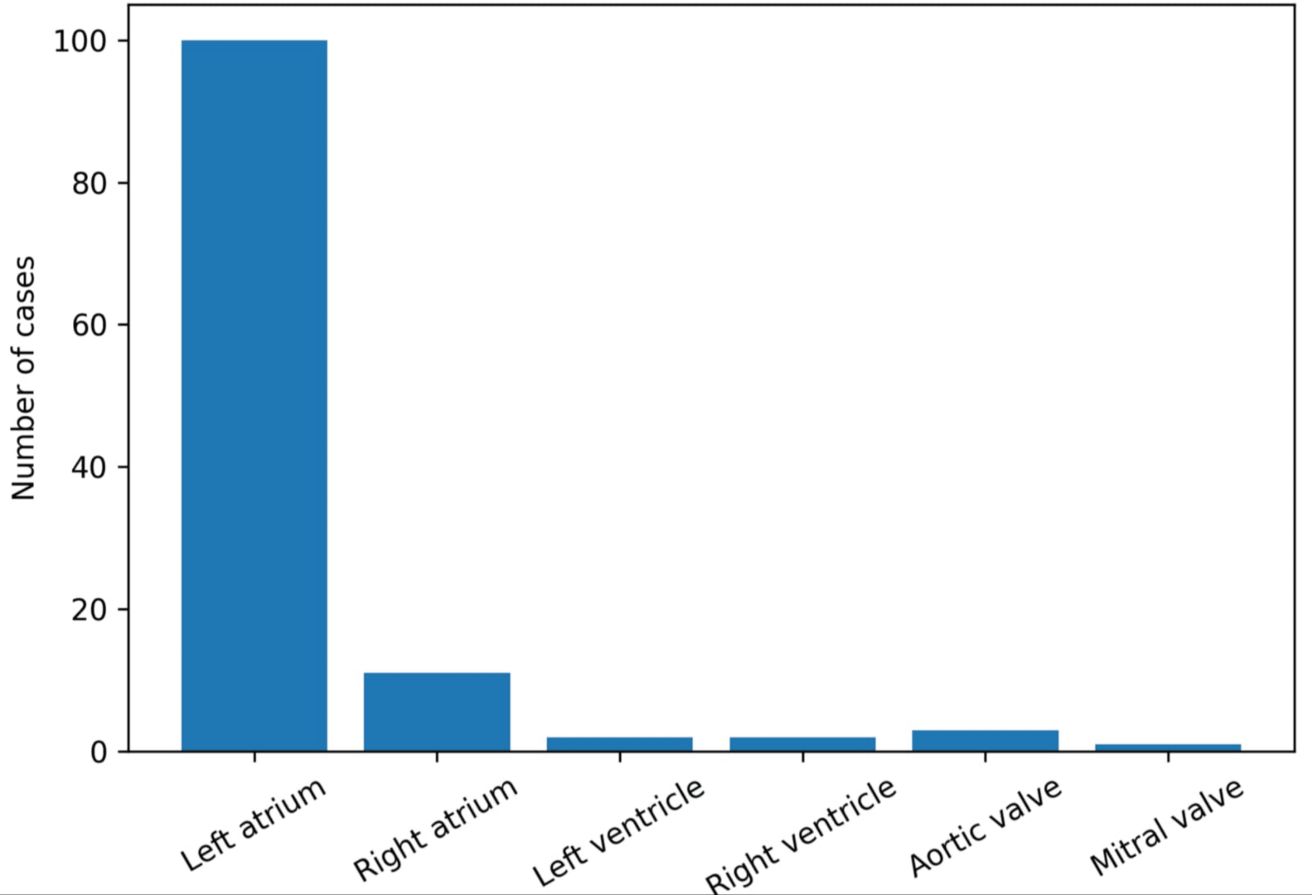
Anatomical distribution of cardiac tumors

Regarding the anatomical location of cardiac neoplasms, the left atrium was the most frequent site, with 100 cases (84%), followed by the right atrium with 11 cases (9.2%). Two cases were identified in both the left ventricle (1.7%) and right ventricle (1.7%). Additionally, three cases (2.5%) were located on the aortic valve and one case (0.8%) on the mitral valve (Figure 2).

**Figure 3:**
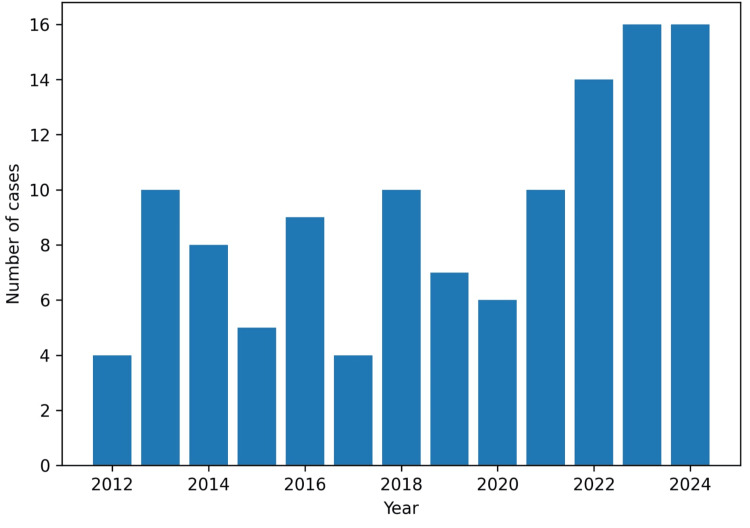
Annual distribution of cardiac tumor cases between 2012 and 2024

The annual distribution showed interannual variations, with a minimum of four cases in 2012 and 2017 (3.4% each) and a maximum of 16 cases in 2023 and 2024 (13.4% each) (Figure 3).

**Figure 4:**
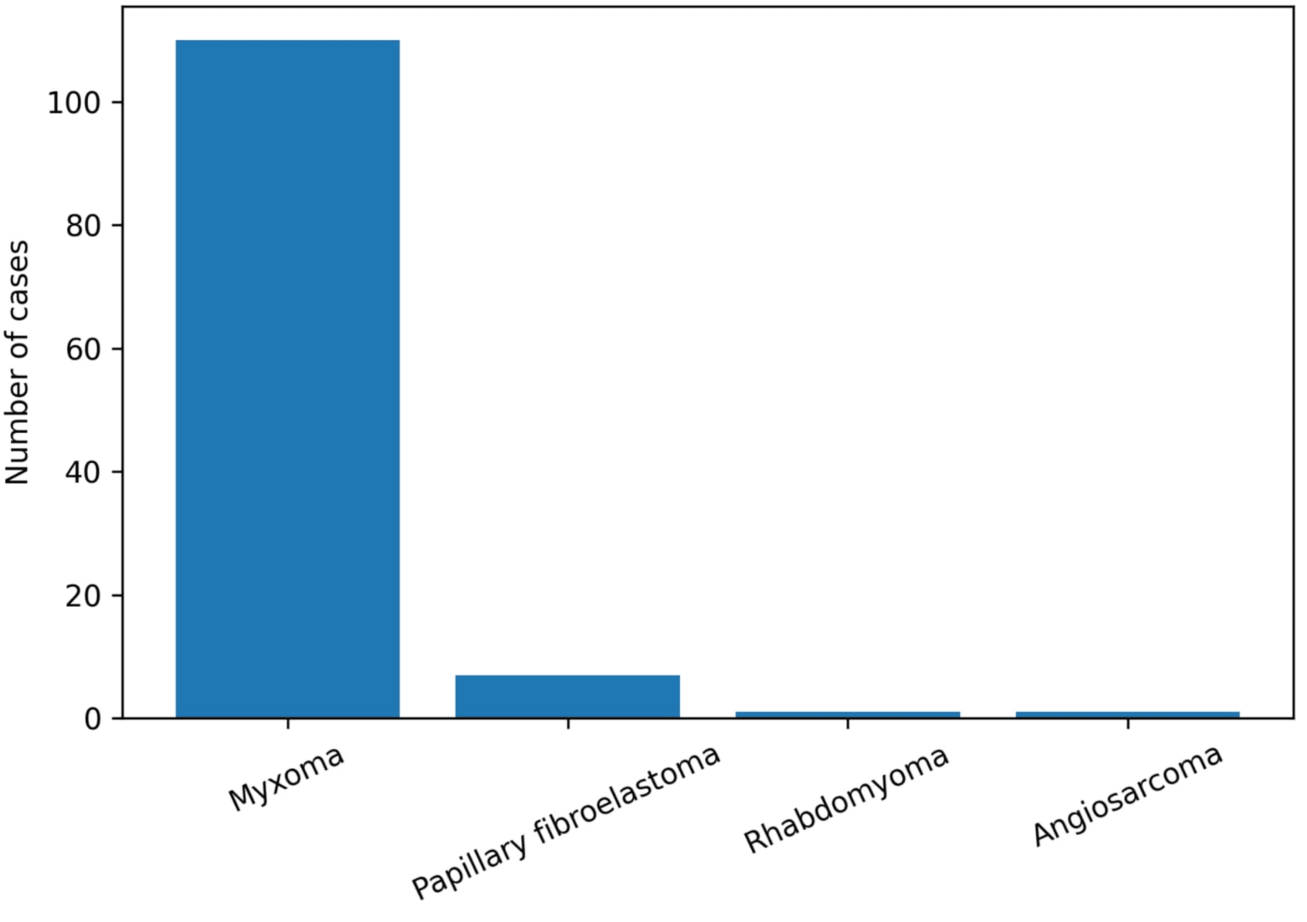
Distribution of cardiac tumors according to histological subtype

Regarding histological subtype, most tumors corresponded to benign neoplasms. Myxoma was the most frequent tumor, with 110 cases (92.4%), followed by papillary fibroelastoma with seven cases (5.9%) and rhabdomyoma with 1 case (0.8%). Among malignant neoplasms, only one case (0.8%) of angiosarcoma was identified. No recurrent cases were identified during the study period in this cohort (Figure 4).

## Discussion

Primary cardiac tumors are uncommon clinical entities, and most available evidence derives from single-center surgical series. The findings of the present study are consistent with previously published reports demonstrating a predominance of benign tumors, particularly atrial myxomas [1]. In this single-center surgical series, benign primary cardiac neoplasms represented the overwhelming majority of cases, with myxoma being the most frequent histological subtype. This distribution aligns with international series, where myxomas account for approximately 70-90% of primary cardiac tumors [2]. This finding is consistent with previous reports describing myxoma as the most frequent primary cardiac tumor in adults, accounting for the majority of benign cardiac neoplasms. The predominance of myxoma in our cohort reinforces the reproducibility of this epidemiological pattern in our population and supports the external consistency of our findings. A predominance of female patients was observed in this study, as has been reported in other cohorts of atrial myxomas. Although the exact mechanism remains unclear, hormonal and biological factors have been proposed as potential contributors to this distribution [8-10]. Malignant primary cardiac tumors remain rare. In the present series, only one case of angiosarcoma was identified, which is consistent with previous reports describing the low frequency of malignant cardiac neoplasms in surgical populations [11]. This finding reflects both the rarity of these tumors and their often aggressive clinical course, which may limit surgical candidacy.

Regarding anatomical distribution, the left atrium was by far the most frequently affected site, followed by the right atrium, whereas ventricular and valvular involvement was uncommon. This pattern closely parallels the known predilection of cardiac myxomas for the left atrium and is in line with previously published surgical and pathological series. From a clinical standpoint, this anatomical predominance is relevant because left atrial tumors are more likely to be identified during the evaluation of obstructive symptoms, embolic phenomena, or incidental imaging findings, and they remain a key diagnostic consideration in patients with intracardiac masses.

Due to the retrospective design of the study, presenting symptoms were not uniformly documented across all records; therefore, a standardized analysis of symptom frequency by tumor location could not be reliably performed. This should be considered when interpreting the clinical relevance of the anatomical findings. Surgical resection continues to be the cornerstone of treatment for primary cardiac tumors. Outcomes are generally favorable in benign tumors, whereas malignant neoplasms are associated with poorer prognosis due to aggressive biological behavior and limited response to adjuvant therapies [12-13]. Institutional series such as this remain valuable, particularly in regions where large multicenter registries are limited. Reporting single-center experiences contributes to a broader understanding of the epidemiological and clinical characteristics of these rare tumors.

Limitations

This study has several limitations. First, its retrospective and single-center design may limit the generalizability of the findings. Second, only patients who underwent surgical resection were included, which may introduce selection bias and exclude patients with unresectable or advanced disease. Third, although the sample size is representative of a single institution, it remains limited due to the rarity of primary cardiac tumors. Finally, long-term follow-up and survival outcomes were limited by the available institutional records, which restricted a more comprehensive outcome analysis beyond the study period.

## Conclusions

Primary cardiac tumors are rare entities, with benign neoplasms, particularly atrial myxomas, accounting for the vast majority of surgically treated cases. The findings of this 12-year institutional experience are consistent with previously reported epidemiological patterns, including a predominance of left atrial involvement and a higher frequency in female patients. This study reinforces the role of surgical resection as the cornerstone of management, with generally favorable outcomes in benign tumors. Additionally, it highlights the rarity of malignant cardiac neoplasms in surgical cohorts. These findings contribute institutional data from a national cardiac referral center in Mexico and reinforce the value of regional evidence from Latin America in characterizing the epidemiological and pathological profile of primary cardiac neoplasms. Future multicenter studies and registries with long-term follow-up are needed to better define outcomes, recurrence patterns, and survival.
